# Focus–Specific Clinical Profiles in Human African Trypanosomiasis Caused by *Trypanosoma brucei rhodesiense*

**DOI:** 10.1371/journal.pntd.0000906

**Published:** 2010-12-07

**Authors:** Lorna M. MacLean, Martin Odiit, John E. Chisi, Peter G. E. Kennedy, Jeremy M. Sternberg

**Affiliations:** 1 Department of Biology, Hull York Medical School, Centre for Immunology and Infection, University of York, York, United Kingdom; 2 Uganda AIDS Commission, Kampala, Uganda; 3 College of Medicine, University of Malawi, Blantyre, Malawi; 4 Department of Neurology, University of Glasgow, Southern General Hospital, Glasgow, United Kingdom; 5 Institute of Biological and Environmental Sciences, University of Aberdeen, Aberdeen, United Kingdom; Institute of Tropical Medicine, Belgium

## Abstract

**Background:**

Diverse clinical features have been reported in human African trypanosomiasis (HAT) foci caused by *Trypanosoma brucei rhodesiense* (*T.b.rhodesiense*) giving rise to the hypothesis that HAT manifests as a chronic disease in South-East African countries and increased in virulence towards the North. Such variation in disease severity suggests there are differences in host susceptibility to trypanosome infection and/or genetic variation in trypanosome virulence. Our molecular tools allow us to study the role of host and parasite genotypes, but obtaining matched extensive clinical data from a large cohort of HAT patients has previously proved problematic.

**Methods/Principal Findings:**

We present a retrospective cohort study providing detailed clinical profiles of 275 HAT patients recruited in two northern foci (Uganda) and one southern focus (Malawi) in East Africa. Characteristic clinical signs and symptoms of *T.b.rhodesiense* infection were recorded and the degree of neurological dysfunction determined on admission. Clinical observations were mapped by patient estimated post-infection time. We have identified common presenting symptoms in *T.b.rhodesiense* infection; however, marked differences in disease progression and severity were identified between foci. HAT was characterised as a chronic haemo-lymphatic stage infection in Malawi, and as an acute disease with marked neurological impairment in Uganda. Within Uganda, a more rapid progression to meningo-encephaltic stage of infection was observed in one focus (Soroti) where HAT was characterised by early onset neurodysfunction; however, severe neuropathology was more frequently observed in patients in a second focus (Tororo).

**Conclusions/Significance:**

We have established focus-specific HAT clinical phenotypes showing dramatic variations in disease severity and rate of stage progression both between northern and southern East African foci and between Ugandan foci. Understanding the contribution of host and parasite factors in causing such clinical diversity in *T.b.rhodesiense* HAT has much relevance for both improvement of disease management and the identification of new drug therapy.

## Author Summary

*T. b. gambiense* and *T. b. rhodesiense* cause human African trypanosomiasis (HAT). These parasite subspecies differ in their rate of progression to central nervous system (CNS) infection, and consequently to coma and death. Variation in disease progression and severity has also been documented between northern and southern East African *T.b. rhodesiense* HAT. However, it is unknown if this is caused by differences in patient susceptibility to infection, genetic variation in parasite virulence, or both, as despite the existence of good molecular tools, previous studies have involved limited numbers of HAT cases. In this paper we present extensive clinical data on *T. b. rhodesiense* cases and describe robust clinical profiles for three disease foci. Common presenting symptoms were identified. We also describe marked differences in disease progression and severity both between Ugandan and Malawi foci, and between two Ugandan foci (Tororo and Soroti) giving rise to three foci-specific clinical phenotypes ranging from a chronic haemo-lymphatic stage infection in Malawi, to rapid disease progression and neurological dysfunction in Soroti and severe neuropathology in Tororo cases. Most importantly, we have now established clinical focus-specific phenotypes that will be available to supplement host and parasite genetics studies to determine their contribution to HAT disease virulence.

## Introduction

Human African trypanosomiasis is caused by infection with African trypanosomes, which are transmitted by the haematophagous tsetse fly. *Trypanosoma brucei gambiense* (*T.b. gambiense*) HAT is present in West and Central Africa and *T.b. rhodesiense* HAT is found in East and Southern Africa. The disease is characterised by two stages, the early or haemo-lymphatic stage where trypanosomes proliferate at the bite site, travel to local lymph nodes and establish infection in the bloodstream, and the late or meningo-encephalitic stage in which trypanosomes invade the central nervous system (CNS) leading to coma and death if untreated [1], [2]. HAT has a severe social and economic impact across sub-Saharan Africa today with an estimated 60 million people at risk of African trypanosome infection and 70,000 people are infected [3].

*T.b. rhodesiense* infection is typically described as an acute disease with rapid progression to late stage infection, however, a wide range of disease pathologies have been reported for many years in East Africa from asymptomatic carriers and mild chronic infections with incubation times of several months in Zambia [4], [5], [6], [7] to accounts of acute infections causing severe disease pathology within 4–6 weeks [8] and 80% of deaths within 6 months [9] in the Busoga focus of Uganda/Kenya. Analysis of historic *T.b. rhodesiense* epidemics in East Africa led to the proposal that HAT spread from Zambia to Tanzania and Uganda increasing in acuteness and virulence towards the north [10]. However, this is unlikely as *T.b. rhodesiense*, not *T.b. gambiense*, is now thought to be responsible for the first recorded HAT epidemic in 1900 which occurred in the Busoga focus [11], [12]. Variation in HAT disease severity between foci suggests that there is genetic variation in trypanosome virulence and/or differences in host susceptibility to trypanosomiasis. Isoenzyme and minisatellite analysis of *T.b. rhodesiense* isolates from the Busoga focus and the Luangwa Valley focus in Zambia have confirmed distinct parasite genotypes between northern and southern East African HAT foci [13], [14], [15]. Correlation of parasite genotypes with different clinical profiles, however, is limited although analysis of a small number of *T.b. rhodesiense* isolates from within the Busoga focus (1989 to 1993) identified two main zymodemes (the ‘busoga’ and ‘zambezi’ zymodemes), which were associated with different clinical manifestations [16]. There is also evidence for differences in host susceptibility to *T.b. rhodesiense* infection. In Zambia different clinical profiles were associated with tribal group and previous HAT exposure [6], [4]. In addition, there are accounts of acute disease pathology in non-local individuals with HAT compared to the typically chronic disease described in Zambian patients [4], [17], [6], [7], [18], [19].

Recently we reported dramatic variation in disease severity between northern and southern East African HAT patients in Uganda and Malawi respectively [20]. HAT patients recruited in Soroti and Tororo foci in East (E) Uganda followed an acute disease course with progression to meningo-encephalitic infection in 87% of patients. In contrast, HAT cases from the Nkhotakota district Central (C) Malawi presented with a mild disease and trypanosome CNS invasion was only diagnosed in 7% of recruits even after several months' incubation. These disease phenotypes were associated with both different host inflammatory cytokine responses and different parasite serum resistance associated (SRA) gene polymorphisms. In addition, differences in disease pathology were also observed between HAT cases recruited in two geographically close foci (Tororo and Soroti) within Eastern Uganda [21]. A higher proportion of Tororo focus HAT cases had progressed to CNS infection, displayed more severe neurological dysfunction and higher plasma IFN-γ production than Soroti focus cases. Trypanosome isolates were found to be genetically distinct, thus, demonstrating that distinct parasite genotypes circulating in the field in spatially separated foci within Uganda are associated with differences in virulence. We also hypothesised that the magnitude of host systemic IFN-γ production determines the post-infection time at which trypanosomes invade the CNS in acute HAT, while the ‘mild’ and ‘severe’ phenotypes specific to northern and southern East African foci, which are geographically much further apart, involve differing abilities to down-regulate TNF-α mediated pathology by TGF-β.

**Figure 1:**
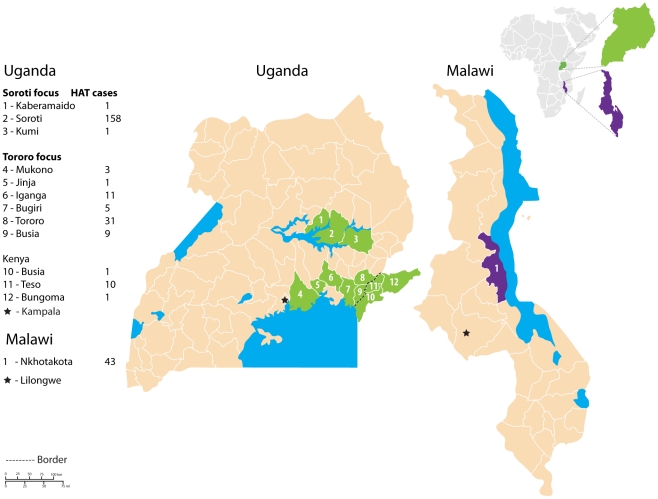
Map of Uganda (green) and Malawi (purple) districts where Sleeping Sickness patients were recruited in this study. The Soroti focus encompasses Uganda districts 1–3 and the Tororo focus encompasses districts 4–12.

These studies suggest that both genetic variation in *T.b. rhodesiense* virulence and differences in host resistance to infection play key roles in HAT disease severity. To examine the influence of parasite and host genetics on HAT severity within and between foci it is essential to determine clinical profiles for HAT in southern and northern East African foci. Here we present an extensive clinical study of *T.b. rhodesiense* HAT in two northern and one southern East African foci (Figure 1). The two northern (Uganda) foci are 150 km apart and are referred to as Tororo and Soroti foci. The Tororo focus is part of the historic Busoga focus, while HAT cases were first detected in the Soroti focus in December 1998 [22], [23]. *G.f. fuscipes* is the vector for transmission in both ‘northern’ foci. The ‘southern’ focus was Nkhotakota district of C Malawi. Sleeping Sickness has been recorded here since 1911 and is it associated with transmission by *G.m. morsitans* and *G. pallidipes* in the Nkhotakota Wildlife Reserve and the Kasungu National Park [24], [25]. We have documented detailed clinical histories of each patient, recorded HAT signs and symptoms on admission, analysed the rate of stage progression, determined parasite burden and CSF WBC counts, and assessed the influence of co-infections on HAT clinical profiles.

## Materials and Methods

### Study sites and patient recruitment

#### Recruitment and diagnosis

This was a site-specific retrospective cohort study. 275 HAT patients were recruited in two northern East African foci (E Uganda and SE Uganda/Kenyan border region), and one southern East African focus (C Malawi) (Figure 1). HAT patient recruitment periods in SE Uganda/Kenyan border focus covered October 1998 to March 1999, June 1999 to September 2000 and July 2002 to July 2003; in E Uganda recruitment began after diagnosis of the first case in this region in December 1998 to March 1999, April 2000 to September 2000, and August 2002 to August 2003; in C Malawi cases were recruited from February 2002 until December 2002. As all HAT cases diagnosed during the defined study periods were recruited (with the exception of 10 cases due to co-infections) they represent an accurate cross section and there is no sample bias. Observation bias is minimised as all individuals are confirmed HAT cases (this is an absolute diagnosis not prone to bias), and clinical parameters are recorded on a standardised patient recruitment form.

During study periods, all HAT patients attended Livestock Health Research Institute (LIRI) hospital SE Uganda (0°42′17″N, 34°10′23″E), Serere Health Centre E Uganda, (1°31′20″N, 33°26′49″E), or Nkhotakota hospital C Malawi (12°57′42″N, 34°14′05″E). These included individuals who self-reported for diagnosis and individuals who had been identified during field surveillance in areas with known tsetse and/or HAT problems. In both cases initial diagnosis involved microscopic examination of wet and thick blood films [26], [27] or the buffy coat region after microhaematocrit centrifugation (mHCT) [28], [29] prepared from finger prick blood. Parasitemia was recorded as the number of parasites observed in 10 fields at 400× magnification.

#### Clinical observations

On admission a comprehensive clinical history was taken and a physical examination performed on all HAT patients. All clinical examinations were performed by a physician (MD) or experienced nurse based at LIRI hospital, Serere Health Centre or Nkhotakota hospital. To ensure consistency in qualitative clinical observations, all staff involved in this study were trained in the use of a standardised patient record form. Vital signs were first assessed including body temperature (fever defined as auricular, oral or axillary temperature of ≥38.0°C), radial pulse (normal adult pulse 60–100 beats per minute) and blood pressure (normal diastolic pressure 60–90 mmHg, normal systolic pressure 110–140 mmHg).

Typical non-neurological signs of HAT [30] were recorded including presence of a chancre, ascites and oedema (severity of oedema categorised as none, mild or severe). The spleen, liver and lymph nodes were palpated and any enlargement recorded. Blood haemoglobin (Hb) was measured to diagnose anaemia by calculating haematocrit levels from packed cell volume (PCV). PCV was measured by collecting peripheral blood into a heparinised capillary tube, centrifuged for 10 minutes at 10,000 rpm in a microhaematocrit centrifuge and the volume of packed cells read as a proportion of the total volume of blood expressed as l/l. This value was converted using the conventional conversion factor: 100 g Hb  = 6.2 mmol Hb  = 0.30 l/l haematocrit [31]. Baseline Hb levels vary with age, gender and pregnancy [32], therefore, we used the criteria by which WHO define mild anaemia and severe anaemia [33]. Briefly, mild anaemia was diagnosed as Hb <110 g/L in children aged 6–59months and pregnant women ≥16 years old; Hb<115 g/L in children aged 5–11years old; Hb<120 g/L in children aged 12–15 years old and non-pregnant women ≥16 years old; Hb<130 g/L in men ≥16 years old. Severe anaemia was diagnosed as Hb ≤60 g/L in all groups.

The Glasgow Coma Score (GCS) [34] was used to assess the level of neurological dysfunction in each HAT case recruited from 2002 onwards. GCS determines best eye opening, motor and verbal response (normal value is 15, and a score of ≤8 indicates severe impairment of consciousness). Other clinical parameters that infer CNS involvement were recorded for all cases such as altered gait (categorised as normal, able to walk if aided or unable to walk), cranial nerve palsies, tremors, urinary incontinence and somnolence [30].

#### HAT staging criteria

Stage of trypanosome infection was determined in accordance with WHO criteria, late stage infection diagnosed when trypanosomes are detected in the cerebrospinal fluid (CSF) or when present in blood and CSF white blood cell (WBC) count >5/mm^3^ [35]. A lumbar puncture was performed on individuals who were either *T. brucei* blood positive or trypanosome blood negative but presenting with symptoms characteristic of late stage HAT [30]. Microscopic examination of CSF using a Neubauer haemacytometer (Hawksley, U.K.) determined CSF parasitaemia and WBC counts. Double centrifugation of CSF was performed in Uganda to increase sensitivity of trypanosome detection [36] in Malawi presence or absence of *T. brucei* in CSF was recorded with WBC counts.

#### Co-infection assessment

285 HAT patients were screened for malaria and filariasis as part of the blood examination protocol. Stool and urine samples were microscopically examined to screen for hookworm infections and schistosomiasis. All individuals diagnosed with malaria, filariasis or schistosomiasis were given treatment but omitted from this study. Cases with hookworm infections were noted but still were recruited. In the Tororo focus, six HAT cases were diagnosed with malaria and one with filariasis; in the Nkhotakota focus two patients presented with malaria co-infection and one with schistosomiasis, therefore, ten HAT cases were omitted from this study.

#### Indicators of infection duration

To evaluate the rate of disease stage progression it was necessary to estimate the duration of trypanosome infection. In each case this was achieved by noting presence of chancre on admission, recording an approximate duration of illness and when bitten according to HAT patients at interview. A hypothetical map of disease progression in each focus was generated by calculating the estimated median post-infection time from patient interview data for all cases presenting with a particular clinical sign or symptom of HAT on admission. A chancre is the first sign of *T.b. rhodesiense* infection, it develops within 5–15 days of inoculation and heals within 2–3 weeks [30], [37], therefore, patients presenting with a chancre had an estimated post-infection time of 19–36 days. Additionally, as a chancre is often accompanied by swollen local lymph nodes, prevalence of localised lymphadenopathy was also used to indicate recent infection.

#### Treatment

The treatment regimen for early and late stage *T.b. rhodesiense* were those used by the National Sleeping Sickness Control programmes. Briefly, in Uganda early stage patients were given Suramin (Antrypol: Bayer: 20 mg/kg) and late stage cases were initially given suramin followed by four series of melarsoprol (MelB) (Arsobal: Rhone-Poulenc: 3.6 mg/kg). In Malawi early stage cases were given four doses of suramin followed by one series of MelB and late stage patients were given two doses of suramin followed by three series of MelB.

### Ethics statement

This study was conducted according to the principles expressed in the Declaration of Helsinki. All patients recruited received written and verbal information explaining the purpose of this study and gave informed consent. The ethical committees in Uganda (Ministry of Health), Malawi (College of Medicine) and the UK (Grampian Joint Ethics Committee) approved all protocols. Ethical consent forms were designed in English and also translated into local languages. Consent was given as a signature or a thumb print after verbal explanation. For those under 16 consent was given by their legal guardian, and for those whose clinical condition prohibited full understanding of the recruitment process, consent was gained from a spouse or other family member. Blood and CSF analysis was carried out on admission and on discharge as part of normal diagnostic and follow-up procedures.

### Statistical analysis

Data analyses were carried out using Statview and JMP (SAS Institute, Cary, North Carolina) statistical packages. The distribution of continuous variables was assessed for normality using the Shapiro-Wilks W test and Levene's test for homogeneity of variance between groups, standard transformations were applied as appropriate [38]. Parametric statistics were used to analyse normally distributed continuous data including the unpaired Student's t test for variation between groups, the paired t test for matched groups and analysis of variance (ANOVA) for comparing more than two groups in conjunction with the Tukey-Kramer post-hoc test. Non-parametric statistics were used when data was not normally distributed and could not be transformed, these included the Mann Whitney U test to compare two groups, the Wilcoxon signed rank test for matched groups and the Kruskal-Wallis test when more than two groups were being compared. Additionally, Spearman's Rho was used to assess relationships between continuous variables and Fisher's exact test was used to test for associations between discontinuous variables. To address missing data, the exact number of cases analysed for each parameter measured is given.

## Results

### HAT patients

#### Geographical distribution

The geographical distribution of 275 HAT patients recruited across three East African foci during several surveillance periods from January 1998 to December 2003 is summarised in Table 1. HAT due to *T.b.rhodesiense* was found in a large number of districts within the historic SE Uganda/Kenyan border (Busoga) focus but the highest number of cases were from Tororo district, thus, this will henceforth be called the Tororo focus. In E Uganda HAT cases were concentrated in Soroti district and so will be called the Soroti focus. Surveillance in C Malawi was restricted to Nkhotakota district in 2002 and will be called the Nkhotakota focus.

**Table 1:** Geographical distribution of HAT patients recruited during 1998–2003.

Focus	Country	District	No. of HAT cases
**Tororo**	Uganda	Tororo	31
		Iganga	11
		Busia	9
		Bugiri	5
		Mokono/Buvuma Island	3
		Jinja	1
	Kenya	Teso	10
		Busia	1
		Bungoma	1
			**Total 72**
**Soroti**	Uganda	Soroti	158
		Kumi	1
		Kaberamaido	1
			**Total 160**
**Nkhotakota**	Malawi	Nkhotakota	43
			**Total 43**

#### Age and gender

The median age of HAT patients (Table 2) did not differ between foci (Kruskal-Wallis test, p>0.05). However, a higher proportion of cases in the Soroti focus were women and children (<16 years old) (Fisher's Exact Test, p≤0.01), while most cases were adult males in the Nkhotakota focus (Fisher's Exact test, p<0.01). This may reflect varying levels of exposure to human-infective trypanosomes due to the different roles within each society for men, women and children. In Soroti women and children traditionally have a cattle-keeping role, while in Nkhotakota many adult males work in the nearby wildlife reserve.

**Table 2:** HAT patient age distribution, gender/age ratio and stage of infection.

HAT Focus	Count	Median age (IQR)[Range]	% adult male: female: child*	Late Stage (%)
Tororo	72	26 (26) [2–75]	43∶39∶18	86^c^
Soroti	160	23 (29) [2–85]	27∶43∶30a, c	77^c^
Nkhotakota	43	28 (19) [1–84]	60^b^∶28∶12	16

*Child <16 years old.

a Significantly higher than Tororo (p<0.05).

b Significantly higher than Soroti (p<0.05).

c Significantly higher than Nkhotakota (p<0.05).

#### *T.b. rhodesiense* infection stage diagnosis

In the Nkhotakota focus HAT patients predominantly presented with haemo-lymphatic stage infections (Table 2), while in the Uganda Soroti and Tororo foci the majority of HAT patients were diagnosed with meningo-encephalitic stage infections (Fisher's Exact Test, p<0.0001).

### Clinical observations

#### Non-neurological characteristic signs of HAT

A high proportion of early stage HAT patients in the Tororo and Soroti foci presented with chancre (Table 3), this was indicative of recent infection (19–36 days). Additionally, in Uganda up to 26% late stage cases presented with chancre demonstrating rapid progression to meningo-encephalitic infection. In the Nkhotakota focus, however, no HAT patients presented with chancre or, according to patient interview information, had ever formed a chancre at the inoculation site.

**Table 3:** Non-neurological signs of HAT in early and late stage infections.

	Clinical observations					
	Tororo focus		Soroti focus		Nkhotakota focus	
**Early stage infections**	**n**	**%**	**n**	**%**	**n**	**%**
Chancre	10	50^ c^	37	46^ c^	36	0
Fever	8	13	37	14	30	50^b^
Raised pulse	2	0	31	10	14	0
Low blood pressure systolic/diastolic	2	100/0	24	21/4	26	0/0
Lymphadenopathy	2	100	26	27	29	10
Splenomegaly	2	0	29	7	31	36^b^
Hepatomegaly	2	0	29	0	30	40^b^
Ascites	2	0	30	0	29	0
Anaemia normal∶mild∶severe	10	10∶80∶10	37	38∶57∶5	25	16∶68∶16
Oedema normal∶mild∶severe	2	50∶50∶0	33	70∶24∶6	32	75∶16∶9
**Late stage infections**	**n**	**%**	**n**	**%**	**n**	**%**
Chancre	61	17^ c^	119	26^ c^	7	0
Fever	58	20	119	14	4	100a, b
Raised pulse	27	11	81	15	6	0
Low blood pressure systolic/diastolic	25	72/28^b^ , c	65	37/8	6	0/0
Lymphadenopathy	23	30	64	48	6	0
Splenomegaly	27	33^b^	69	15	6	0
Hepatomegaly	25	16^b^	67	3	6	0
Ascites	27	4	78	9	5	0
Anaemia normal∶mild∶severe	62	14∶81∶5	123	4∶91^a^∶5	4	25∶75∶0
Oedema normal∶mild∶severe	26	85∶15∶0	76	42∶47^a^∶11	6	67∶33∶0

n represents the number of patients for which presence or absence of each clinical observation was recorded, prevalence of each parameter is expressed as a percentage of n.

a Significantly higher than Tororo (p<0.05).

b Significantly higher than Soroti (p<0.05).

c Significantly higher than Nkhotakota (p<0.05).

Fever was a common presenting symptom in both early and late stage *T. b. rhodesiense* infections in the Malawi Nkhotakota focus but was less frequently observed in Ugandan patients (Fisher's Exact Test, p≤0.001) (Table 3). However, in interview, the prevalence of recent fever was extremely high in Ugandan HAT cases suggesting intermittent waves of fever. Although raised radial pulse was not observed in Nkhotakota cases and infrequently in Ugandan cases, other signs of cardiovascular involvement were commonly recorded in Ugandan *T.b. rhodesiense* HAT. Low systolic and diastolic blood pressure were significantly more prevalent in late stage Tororo HAT patients than those in Soroti and Nkhotakota foci (Fisher's Exact Test, p<0.05), while mild oedema was more frequently recorded in Soroti late stage cases than those in Tororo (Fisher's Exact Test, p = 0.01) (Table 3).

Abdominal palpation revealed splenomegaly and hepatomegaly were common signs of HAT in Nkhotakota focus early stage HAT patients, this was particularly so in children (<16 years old) (Fisher's Exact Test, p<0.05). However, prevalence of both was significantly lower in the Soroti focus (Fisher's Exact Test, p≤0.01), and not observed in the small number of early stage Tororo cases. In late stage HAT, however, splenomegaly and hepatomegaly were significantly more frequently observed in Tororo than Soroti focus cases (Fisher's Exact Test, p<0.05) (Table 3). Occurrence of hepatomegaly correlated with splenomegaly in Sleeping Sickness patients in all foci (Fisher's Exact Test, p<0.0001). Ascites was only observed in a small number of late stage HAT cases in Ugandan foci.

Mild anaemia was the most common presenting clinical sign of both early and late stage HAT in all foci (Table 3). Mild anaemia was more prevalent in late stage cases in the Soroti focus compared to Tororo focus cases (Fisher's Exact Test, p  = 0.01). Severe anaemia was not frequently diagnosed, but was most prevalent in early stage patients in the Nkhotakota focus.

#### Neurological signs characteristic of HAT

A rapid onset of neurological dysfunction was observed in Soroti haemo-lymphatic stage HAT cases. Comparison of prevalence of neurological signs of HAT between early stage Soroti and Nkotakota cases revealed Soroti focus cases displayed a higher prevalence of altered gait on admission (Fisher's Exact Test p<0.05), tremors (Fisher's Exact Test, p<0.001), cranial nerve palsies (Fisher's Exact Test, p<0.01), urinary incontinence and somnolence (Fisher's Exact Test, p<0.0001) (Table 4). It was not possible to compare Tororo focus early stage HAT due to the small group size.

**Table 4:** Neurological signs & mortality rates in early and late stage HAT infections.

	Clinical observations						
	Tororo focus		Soroti focus			Nkhotakota focus	
**Early stage infections**	**n**	**%**		**n**	**%**	**n**	**%**
Altered Gait normal∶aided∶unable	2	100∶0∶0		33	36∶46^c^∶18	28	65∶21∶14
Tremors	2	0		33	61^c^	30	17
Cranial neuropathies	2	0		33	30^c^	31	3
Urinary Incontinence	2	0		31	16	30	7
Somnolence	2	0		33	58^c^	25	4
GCS <15	2	0		33	12	32	3
GCS ≤8	2	0		33	0	32	0
Mortality	10	0		37	0	36	6
**Late stage infections**	**n**	**%**		**n**	**%**	**n**	**%**
Altered Gait normal∶aided∶unable	26	54∶31∶15		83	47∶35∶18	6	50∶17∶33
Tremors	25	0		84	67^a^	6	33
Cranial neuropathies	26	0		81	31^a^	6	17
Urinary Incontinence	27	26		83	17	6	17
Somnolence	26	46		84	54	4	25
GCS <15	27	52^b^		84	14	6	33
GCS ≤8	27	10^b^		84	3	6	3
Mortality	62	11		123	6	7	14

n represents the number of patients for which presence or absence of each clinical observation was recorded, prevalence of each parameter is expressed as a percentage of n.

a Significantly higher than Tororo (p<0.05).

b Significantly higher than Soroti (p<0.05).

c Significantly higher than Nkhotakota (p<0.05).

In meningo-encephalitic stage cases cranial neuropathies and tremors were more prevalent in Soroti than Tororo focus cases (Fisher's Exact Test, p = 0.001, p<0.0001 respectively), while abnormal GCS was most frequently recorded in Tororo focus patients (Fisher's Exact Test, p<0.001) (Table 4). In addition, a higher prevalence of coma was observed in Tororo than Soroti focus late stage patients (Fisher's Exact Test, p<0.05). The proportion of patients presenting with altered gait, somnolence and urinary incontinence continued to be high in Soroti but prevalence in Tororo and Nkhotakota foci increased, therefore, no significant differences in these clinical parameters was detected between foci.

Interestingly, a higher prevalence of signs of neurological involvement was recorded in children in Uganda HAT foci. Altered gait was more frequently observed in early stage patients under 16 in the Soroti focus and in late stage Tororo focus cases (Fisher's Exact Test, p≤0.02), urinary incontinence was also more prevalent in children with late stage infections in the Tororo focus (Fisher's Exact Test, p<0.05), and severe neurological dysfunction (GCS ≤8) was more commonly recorded in children with late stage infections in both Uganda foci (Fisher's Exact Test, p<0.05), in fact all cases presenting with GCS ≤8 in the Tororo and Soroti foci were male and ≤12 years old.

#### Mortality

Mortality rates did not significantly differ between HAT foci (Table 4) and there was no correlation with patient age. In Nkhotakota one of the early stage fatalities occurred after developing bacterial meningitis, the other occurred after HAT treatment of an unknown cause, while one meningo-encephalitic stage case died after two doses of MelB. In Uganda four late stage patients (2 Tororo, 2 Soroti) died before MelB was administered, three of which were recruited with severe neurodegeneration (GCS 9–13). However, seven late stage HAT cases (4 Tororo, 3 Soroti) died during or just after the first two series of MelB treatments suggesting possible arsenical encephalopathy [30], and a further two patients died after treatment. There is no information on time of death for one Soroti case. The clinical symptoms found to be significantly associated with fatal outcome across foci included oedema, altered gait, urinary incontinence and abnormal GCS (Fisher's Exact Test, p<0.001, p<0.001, p<0.05 & p = 0.001 respectively).

### Common clinical observations in *T.b. rhodesiense* infection

To establish which clinical observations were indicative of early and late stage *T.b. rhodesiense* infection the most frequently observed signs and symptoms in each focus (present in more than one third of all HAT patients examined on admission) are summarised in Table 5. Mild anaemia was commonly diagnosed in both haemo-lymphatic stage and meningo-encephalitic stage *T.b. rhodesiense* infections in all three foci. In both Ugandan foci chancre was a frequent presenting sign of early stage HAT, however, Soroti early stage HAT was characterised by tremors, somnolence and walking only when aided pointing to early onset neuropathology, while Nkhotakota early stage HAT was characterised by hepatomegaly, splenomegaly and fever. Although somnolence was indicative of late stage HAT in both Ugandan foci only, severe neurological dysfunction was particularly prevalent in Tororo focus HAT. Clear differences in HAT disease profiles, therefore, were observed between distant HAT foci and those within close proximity.

**Table 5:** The most prevalent (in >1/3 cases) clinical observations in *T.b. rhodesiense* HAT.

Early stage HAT characteristics			Late stage HAT characteristics		
Clinical observation	n	%	Clinical observation	n	%
**Tororo focus**			**Tororo focus**		
Lymphadenopathy	2	100	Mild anaemia	64	81
Low systolic BP	2	100	Low systolic BP	25	72
Mild anaemia	10	80	GCS<15>8	27	52
Mild oedema	2	50	Somnolence	26	46
Chancre	10	50			
**Soroti focus**			**Soroti focus**		
Tremors	33	61	Mild anaemia	122	91
Somnolence	33	58	Tremors	84	67
Mild anaemia	37	57	Somnolence	84	54
Chancre	37	46	Lymphadenopathy	64	48
Gait aided	33	45	Mild oedema	76	47
			Low systolic BP	65	37
			Gait aided	83	35
**Nkhotakota focus**			**Nkhotakota focus**		
Mild anaemia	25	68	Fever	4	100
Fever	30	50	Mild anaemia	4	75
Hepatomegaly	30	40			
Splenomegaly	31	36			

### *T.b. rhodesiense* infection duration and rate of stage progression

#### Estimated duration of infection

According to HAT patient interview data (Table 6) early stage cases in the Nkhotakota focus had been infected for longer (median 30 days) than Soroti focus patients (median 23 days) (ANOVA, p = 0.01). Furthermore, after an estimated post-infection time of ≥90 days a significantly larger proportion of Nkhotakota cases had not progressed to late stage infection (Fishers Exact test, p<0.01) suggesting prolonged haemo-lymphatic infection in this focus. Most rapid progression to late stage infection occurred in Soroti focus HAT patients (Table 6), this was significantly shorter than both in Tororo focus patients and Nkhotakota focus patients (ANOVA, p<0.001 & 0.01 respectively), and was supported by a higher prevalence of chancre in Soroti focus late stage cases. Furthermore, a significantly higher percentage of Tororo late stage cases had been infected for ≥90 days than observed in Soroti (ANOVA, p<0.01). Thus, *T.b. rhodesiense* infection in the Nkhotakota focus did not cause chancre formation and progression to CNS infection occurred on average 5 months after infection. Within Uganda, however, HAT followed a more aggressive disease course. This was particularly apparent in Soroti focus cases where meningo-encephaltic stage infection occurred in less than 2 months.

**Table 6:** Estimated HAT infection duration and rate of stage progression using patient interview data.

HAT Focus	Estimated post-infection time DaysMedian (IQR) [Range]	Estimated infection duration ≥90 days (%)
**Early stage**		
Tororo n = 10	26 (43) [5–120]	12
Soroti n = 37	23 (26) [2–90]	3
Nkhotakota n = 36	30 (99) [10–330]^b^	33
**Late stage**		
Tororo n = 62	82 (111) [13–366]^b^	47^b^
Soroti n = 123	50 (53) [6–240]	24
Nkhotakota n = 7	135 (150) [30–240]^b^	67

a Significantly higher than Tororo (p<0.05).

b Significantly higher than Soroti (p<0.05).

#### Disease progression

**Figure 2:**
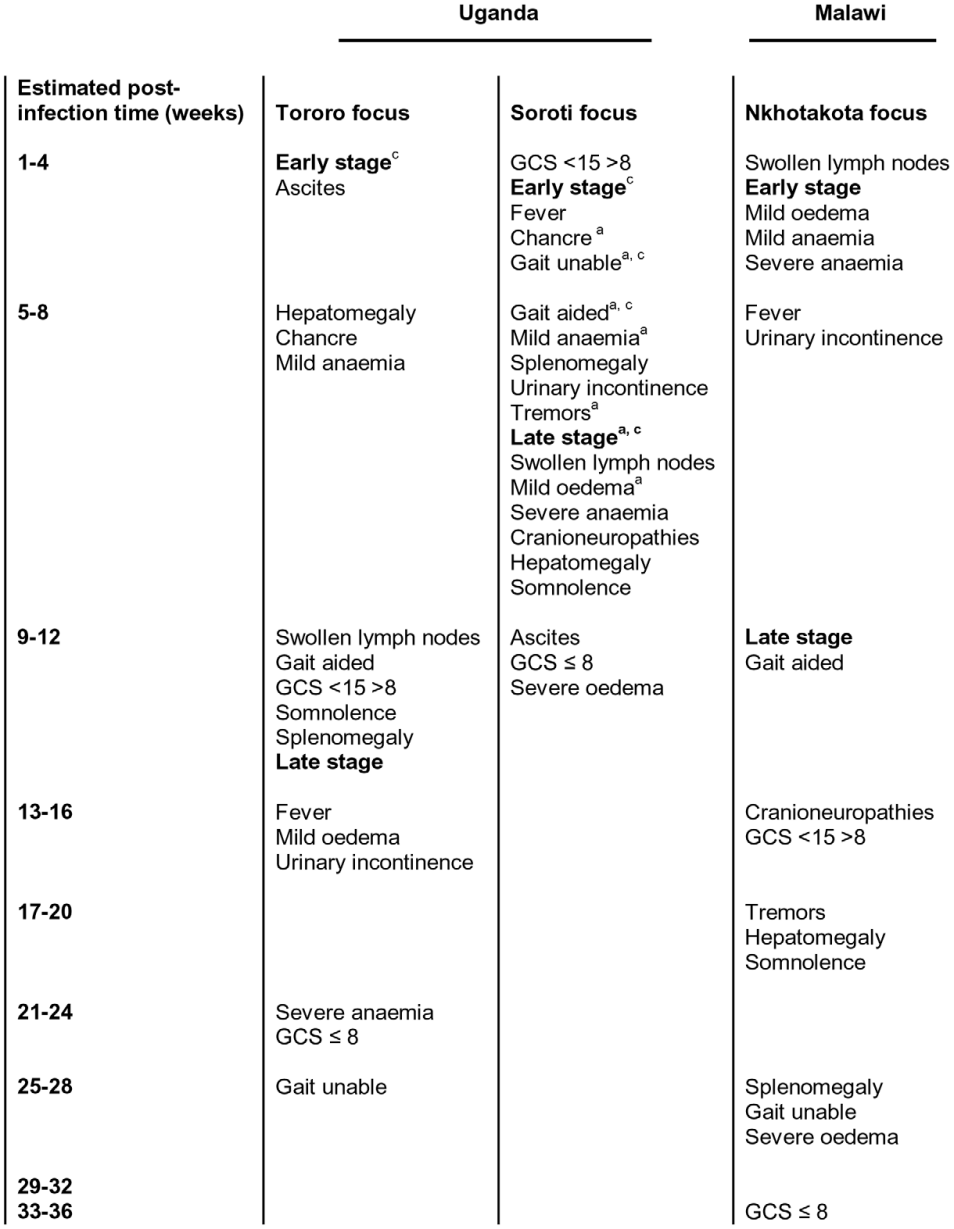
Map of *T.b. rhodesiense* HAT disease progression. Estimated post-infection time (weeks), from patient interview data, at which each clinical observation was recorded in Tororo, Soroti and Nkhotakota foci HAT patients on admission. a. Significantly earlier than Tororo (p<0.05). b. Significantly earlier than Soroti (p<0.05). c. Significantly earlier than Nkhotakota (p<0.05).

The estimated duration of *T.b. rhodesiense* infection, according to patient interview information, has been used to map the time at which each clinical HAT symptom recorded was present in Tororo, Soroti and Nkhotakota foci patients thereby generating a summary of *T.b. rhodesiense* HAT disease progression as schematically represented in Figure 2.

Non-neurological signs of Sleeping Sickness generally manifested at an earlier post-infection time in Soroti focus patients than Tororo focus cases (Figure 2) These included chancre (Student's t-test, p<0.05), mild anaemia (ANOVA, p = 0.001) and mild oedema (ANOVA, p<0.05). Furthermore fever, swollen lymph nodes, splenomegaly and severe anaemia were all observed in Soroti focus cases before Tororo HAT patients but post-infection times did not significantly differ. In Nkhotakota focus patients the observation times of non-neurological signs of HAT did not significantly differ from Ugandan foci, however, splenomegaly and hepatomegaly manifested at noticeably later post-infection times in Malawi and was significantly correlated with haemo-lymphatic stage infection duration (Mann Whitney U test, p<0.01).

Signs of neurological dysfunction were observed earliest in Soroti focus HAT patients. In particular, altered gait was observed at an earlier post-infection time than Tororo and Nkhotakota HAT patients (ANOVA, p<0.05 & 0.01 respectively). Tremors were also observed in Soroti focus cases before Nkhotakota HAT patients (Student's t-test, p = 0.01) and were not observed in Tororo focus cases. All other neurological signs of HAT recorded (cranial neuropathies, urinary incontinence, somnolence, abnormal GCS) were also observed first in Soroti focus HAT patients but this did not significantly differ from other foci. In addition, cases with a fatal outcome had shorter infection duration times in Ugandan foci (both 90 days) than Nkhotakota focus cases (195 days).

This data establishes three different disease progression profiles for *T.b. rhodesiense* HAT indicative of marked differences both between northern and southern East African foci and between Ugandan foci. *T.b. rhodesiense* HAT follows a particularly acute disease course in the Soroti focus with CNS involvement apparent very early in infection and more rapid progression to late stage infection. In Tororo, progression to late stage infection was slower than in Soroti, however, late stage HAT patients manifested with the most severe neurological dysfunction Furthermore, HAT patients in the Tororo focus presented with neurological symptoms before Nkhotakota cases where a chronic disease course was observed characterised by a prolonged haemo-lymphatic stage of infection associated with hepato- and splenomegaly.

### Parasite burden

Parasitaemia (Table 7) did not significantly differ in early stage HAT cases between Soroti and Tororo Ugandan foci (no parasitaemia data was available for Nkhotakota focus cases). Parasitaemia was significantly higher, however, in early stage compared to late stage infections in both Ugandan foci (Mann Whitney U test, p<0.05 & 0.01 respectively). CSF parasite levels did not significantly differ between Ugandan foci (no data was available for Nkhotakota cases). CSF WBC counts were significantly elevated in Tororo late stage cases compared to Soroti patients (Mann Whitney U test, p<0.0001, (Nkhotakota cases also presented with higher CSF WBC counts but this was not significant due to the low number of late stage cases). CSF WBC count was positively correlated to CSF parasite levels in late stage HAT cases in Tororo (Spearman's Rho 0.312, p<0.05) and Soroti foci (Spearman's Rho 0.532, p<0.0001), however, only high CSF parasite levels was found to be associated with a fatal outcome in both Ugandan foci (Mann Whitney U test, p<0.01).

**Table 7:** Blood parasitaemia, CSF parasite levels and CSF WBC counts in HAT patients.

HAT focus	Wet film parasitaemiaper 10 fields (400x)Median (IQR) [Range]		CSF Parasite loadper mm^3^Median (IQR) [Range]		CSF WBC countper mm^3^Median (IQR) [Range]	
**Early stage**	**n**					
Tororo	10	13 (40) [0–840]^d^				
Soroti	11	3 (82) [0–400]^d^				
Nkhotakota	0	ND				
**Late stage**	**n**		**n**		**n**	
Tororo	56	0 (3) [0–40]	49	1 (5) [0–69]	58	74 (110) [6–704]^b^
Soroti	52	1 (6) [0–400]	109	2 (5) [0–1440]	116	35 (67) [2–938]
Nkhotakota	0	ND	0	ND	4	81 (193) [248]

n represents the number of HAT patients for which parasite burden and CSF WBC counts were recorded.

b Significantly higher than Soroti (p<0.05).

d Significantly higher than late stage infection (p<0.05).

### Prevalence of co-infections

HAT cases with malaria, schistosomiasis and filariasis were excluded from this study, however, those with hookworm co-infection were recruited. Hookworm co-infection was not observed in the Nkhotakota focus and only 1% of Soroti focus HAT cases presented with hookworm infection. In Tororo focus HAT cases hookworm was not highly prevalent (13%), however, it was more frequently observed than in other foci (Fishers Exact Test, p<0.0001). Hookworm co-infection was not correlated with HAT stage of infection, infection duration or fatal outcome and did not account for the high prevalence of anaemia observed in HAT cases (Table 3).

## Discussion

The presenting clinical signs and symptoms of *T.b. rhodesiense* HAT can be relatively non-specific, however, typically the first sign of infection is a local acute skin inflammatory response called a trypanosomal chancre at the inoculation site which appears within 5–15 days, is usually 2–5 cm in diameter [37] and lasts for 2–3 weeks. Haemo-lymphatic system involvement begins 1–3 weeks after infection and is typically accompanied by fever, lymphadenopathy, hepatomegaly, splenomegaly, skin rash, pruritis, tachycardia, weight loss, general malaise, and weakness [30], [39]. The meningo-encephalitic stage of infection commences when trypanosomes cross the blood brain barrier (BBB) and invade the CNS. It is associated with psychiatric, motor and sensory disorders, and the classic reversal of sleep patterns leading eventually to coma and death [1]. The transition from early to late stage in *T.b. rhodesiense* infection can occur within a few weeks, indicative of an acute disease, often before neurological involvement becomes pronounced [40], [41]. This ‘typical’ disease profile for *T.b. rhodesiense* HAT, however, varies enormously between foci. In this study of 275 *T.b. rhodesiense* HAT cases in three East African foci (Tororo and Soroti foci, E Uganda; Nkhotakota focus, C Malawi) mild anaemia was the only common (>1/3 cases) presenting clinical observation. Anaemia is a characteristic feature of bovine trypanosomiasis [42] and experimental *T. brucei* infections in mice [43], however, in HAT anaemia has generally not been recorded as one of the main symptoms of human *T.b. rhodesiense* infection [30], [44] or has been attributed to other parasitic infections or malnutrition [45], [46], [47]. The high rate of anaemia in HAT cases in Malawi (including a subset of the Nkhotakota patients in this study) has been previously reported [48]. Marked differences in other HAT presenting signs and symptoms between study sites allowed us to establish *T.b. rhodesiense* foci-specific clinical profiles (Table 5).

Early stage HAT cases in two Ugandan foci typically presented with a chancre, in contrast, none of the Nkhotakota focus patients developed a lesion at the tsetse fly bite site (Table 3). Lack of chancre development may be associated with lower parasite virulence as it occurs more frequently in *T.b. rhodesiense* infections than the more chronic *T.b. gambiense* disease [49], [2], however, it may also be associated with patient ethnicity and previous exposure to *T.b. rhodesiense* as chancre has been more commonly reported in European cases with both *T.b. rhodesiense* and *T.b. gambiense* infections [50], [44], [7], [49], [4]. Instead Nkhotakota early stage HAT cases typically presented with fever, splenomegaly and hepatomegaly. Splenomegaly, caused by increased inflammation with an accumulation of activated macrophages in splenic sinusoids, is described as a symptom characteristic of both early stage *T.b. rhodesiense* and *T.b. gambiense* HAT [39], [30]. The high prevalence of hepatosplenomegaly in haemo-lymphatic stage Nkhotakota cases (Table 3 & 5), similar to that observed in *T.b. gambiense* infections [49], [41], could indicate a more pronounced immune response to *T.b. rhodesiense* infection, which may be associated with improved control leading to the chronic disease course typically observed in this focus. This would be consistent with observations of pathogenesis in malaria where spleen size and the rate of splenomegaly increase with age and immunity, previous exposure to *P. falciparum* can alter spleen response in malaria, and splenomegaly is associated with both protection [51] and a better prognosis in cerebral malaria [52].

The effects of *T.b. gambiense* infection on the cardiovascular system have been well described, with a high prevalence of electrocardiogram (ECG) changes recorded [53]. Less is known in *T.b. rhodesiense* HAT, however, there is some evidence for ECG alterations during infection [54], histological examinations have shown myocardinal degeneration and interstitial haemorrhage [55], and infectious perimyocarditis can be more severe which can lead to a fatal outcome [55], [56]. Indeed, in some studies, cardiac involvement is thought to cause death before neurological deterioration manifests [40], [41]. Previously recorded symptoms of cardiac involvement include tachycardia [39] and generalised oedema, especially of the extremities and the face, caused by increased capillary fragility and permeability [57], which has been associated with congestive heart failure in *T.b. rhodesiense* HAT [58]. In this study, tachycardia was rarely recorded in haemo-lymphatic stage cases, although generalised mild oedema was highly prevalent in late stage Soroti cases (Table 3 & 5). In addition, low systolic blood pressure was frequently observed in late stage HAT cases in both Ugandan foci, which can lead to organ failure. However, in Soroti the haemo-lymphatic stage HAT was uniquely distinguished by very early onset neurological involvement (Table 4 & 5). Motor system involvement was frequently observed in Soroti early stage cases presenting as extrapyramidal disorders such as limb and tongue tremors, cerebellar ataxia leading to alteration in gait, and characteristic sleep disturbances including uncontrollable urges to sleep and classic reversal of the sleep-wake cycle with daytime somnolence alternating with nocturnal insomnia [59], [44], [30]. This is particularly interesting as neurological dysfunction has not previously been clearly documented in African HAT cases prior to detection of trypanosomes in the CSF. In 84% of the early stage Soroti focus HAT cases, for which we have recorded neurological observations (Table 4), CSF double-centrifugation was performed before CSF microscopic examination thereby increasing the sensitivity of parasite detection [27] both on admission and discharge. All early stage cases were blood and CSF negative for parasites after suramin treatment, and CSF WBC counts were ≤5/mm^3^ in all cases on discharge with the exception of one patient (CSF WBC 6/mm^3^) suggesting these were indeed haemo-lymphatic stage infections and not misdiagnosed late stage cases. In Uganda HAT cases are followed up at 3, 6, 12 and 24 months but the follow up rate is less than half. Unfortunately we do not have follow-up records for these specific cases, however, patients are only discharged if neurological features are absent, and it is exceptional to receive patients returning with relapsed infections and there is no empirical evidence to indicate that there are deaths in the community that may be associated with relapses (M.Odiit, Unpublished observations). In *T.b. gambiense* early stage patients with neurological signs would be treated with the late stage drug regimen due to the high relapse rates when treated with suramin and pentamidine [60]. CNS involvement has been previously reported in early stage HAT infections in non-African patients [44], suggesting host genotype/inflammatory response plays a role in the onset of neurological dysfunction. Unfortunately the low number of Tororo focus haemo-lymphatic stage HAT cases did not allow us to establish a robust early stage clinical profile for this focus.

Meningo-encephalitic stage *T.b. rhodesiense* infection is typically associated with severe neurological deterioration, leading to somnolence, coma and death. This was most apparent in the Tororo focus where late stage cases displayed severe neurological dysfunction on admission. This was assessed using GCS, which measures alterations in patient motor, verbal and eye opening responses [34]. Abnormal GCS and somnolence were characteristic symptoms of late stage HAT in Tororo (Table 4 & 5). Soroti late stage HAT cases also commonly presented with symptoms indicative of neurological dysfunction including somnolence, tremors and walking only if aided (Table 4 & 5). In late stage Nkhotakota cases although tremors, altered gait and somnolence were recorded in a third or less cases, the number of late stage cases recruited in this focus were low.

Distinct differences in duration of infection and the rate of stage progression in HAT were also established between patients from foci in Uganda and Malawi, and between cases from different Ugandan foci. In the Nkhotakota focus of Malawi *T.b. rhodesiense* HAT manifested as a chronic long-term disease. Overwhelmingly HAT patients presented with haemo-lymphatic stage infections (Table 2), no chancre formed at the bite site and regional lymphadenopathy were rarely observed (both signs characteristic of early infection). We also used patient interview data to estimate post-infection times. Although interview data can be inaccurate and subject to bias, it is the only method available in field studies of HAT, and was supported both by disease stage diagnosis and clinical indicators of early infection. This data showed estimated early stage infection duration was significantly longer in Nkhotakota cases than observed in the Soroti focus, with a third infected for more than 3 months without progressing to meningo-encephalitic stage infection (Table 6). Furthermore, prolonged haemo-lymphatic stage infection in Nhkotakota was associated with splenomegaly. These observations are more characteristic of *T.b. gambiense* infections and consistent with the previously described chronic *T.b. rhodesiense* HAT in Zambia [4], [5], [6], [7]. In contrast, *T.b rhodesiense* HAT followed a more acute disease course in Uganda. The prevalence of meningo-encephalitic stage infections was extremely high in both Ugandan foci (Table 2) despite shorter estimated infection duration periods than Nkhotakota cases (Table 6). This rapid progression to late stage infection in Uganda was supported by the presence of chancre and regional lymphadenopathy in late stage cases in both Ugandan foci (Table 3) in conjunction with signs and symptoms of severe neurological involvement (Table 4). Differences in disease progression between Ugandan foci, only 150 km apart, were also apparent, with Tororo late stage cases estimated to have been infected for a significantly longer period of time than Soroti cases (Table 6). This would suggest that *T.b. rhodesiense* penetration of the CNS is a more rapid process in Soroti than Tororo HAT cases, however, these infection duration differences must be interpreted with some caution. Previous work analysing a subset of Soroti and Tororo HAT cases presented here did not show any significant difference in infection duration between these foci [21]. Comparison of blood and CSF parasite levels (Table 7) between Ugandan foci did not significantly differ suggesting distinct HAT disease phenotypes established were not due to parasite burden. However, CSF WBC counts were significantly higher in Tororo late stage cases than those from the Soroti focus (Table 7), which may be indicative of a better host immune response to infection in Tororo cases. In addition, to determine robust clinical profiles in natural *T.b. rhodesiense* infections it is important to consider the impact of co-infections, thus, HAT cases with highly prevalent diseases such as malaria, filariases or schistosomiasis were not recruited, and those with hookworm infections showed no association with anaemia, HAT severity or progression. However, it is also important to mention human immunodeficiency virus (HIV) prevalence. By the end of 2003, 14% of the adult population in Malawi and 4% of the adult population in Uganda were infected with an estimated 84,000 and 78,000 annual deaths from acquired immune deficiency syndrome (AIDS) respectively [61]. HAT patient HIV status was not determined in this study, therefore, it cannot be ruled out as a confounding factor in HAT disease severity. However, the higher HIV prevalence recorded in Malawi, where HAT is characterised as a more chronic disease than Uganda, at this time suggests HAT focus-specific clinical phenotypes are not influenced by HIV co-infection.

### Concluding remarks

Overall, a comparison of clinical HAT profiles between foci established marked differences in disease severity between Uganda and Malawi demonstrating that *T.b. rhodesiense* HAT manifests as a chronic disease in Malawi with a low prevalence of neurological symptoms. Within Uganda, HAT is an acute disease with marked neurological involvement contrasting with the clinical profiles previously documented in SE Uganda. In addition, in Soroti a more aggressive disease course was observed with extremely early onset neurological involvement, although neurological deterioration associated with the final stages of HAT were more frequently observed in late stage patients in the Tororo focus.

Establishing distinct clinical phenotypes in different HAT foci can improve disease management by focusing control and surveillance in regions where human disease progression and severity are most extreme. It is now critical to determine the mechanisms involved in causing this clinical diversity.

## Supporting Information

Checklist S1 STROBE checklist cohort.
(0.09 MB DOC)
